# Timeliness of information disclosure during the low transmission period of COVID-19: resident-level observational study in China

**DOI:** 10.1186/s12889-022-12804-x

**Published:** 2022-03-01

**Authors:** Tingting Yang, Xin Shen, Yongguang Yang, Yong Gan, Jing Feng, Zihui Lei, Weixin Zhang, Yuxin Zhao, Lijun Shen

**Affiliations:** 1 Department of Nutrition, Henan Provincial People’s Hospital and Zhengzhou University People’s Hospital, Zhengzhou, Henan China; 2 Department of Social Medicine and Health Management, School of Public Health, Tongji Medical College, Huazhong University of Science and Technology, Wuhan, Hubei China; 3 Department of Research Management, Henan Provincial People’s Hospital and Zhengzhou University People’s Hospital, Zhengzhou, Henan China; 4 School of Public Health, Jilin University, Changchun, Jilin China; 5 Community Health Service Management Center, Shenzhen Fuyong People’s Hospital, Shenzhen, Guangdong China; 6 Department of Clinical Research Center, Henan Provincial People’s Hospital and Zhengzhou University People’s Hospital, No. 7, Weiwu Road, Jinshui District, Zhengzhou, 450003 Henan China

**Keywords:** Information disclosure, COVID-19, Information, China, Disclosure, Low transmission period, Public attitudes, Health information

## Abstract

**Background:**

Only when people feel they have received timely disclosure will they have sufficient incentive to implement community prevention and control measures. The timely and standardized information published by authorities as a response to the crisis can better inform the public and enable better preparations for the pandemic during the low transmission period of COVID-19; however, there is limited evidence of whether people consent that information is disclosed timely and influencing factors.

**Methods:**

A cross-sectional survey was conducted in China from 4 to 26 February 2021. Convenient sampling strategy was adopted to recruit participators. Participants were asked to filled out the questions that assessed questionnaire on the residents’ attitudes to information disclosure timely. A binary logistic regression analysis was performed to identify the risk factors affecting the residents’ attitudes.

**Results:**

A total of 2361 residents filled out the questionnaire. 1704 (72.17%) consented COVID-19 information has been disclosed timely. Furthermore, age (OR = 0.093, 95%CI = 0.043 ~ 0.201), gender (OR = 1.396, 95%CI = 1.085 ~ 1.797), place of residence (OR = 0.650, 95%CI = 0.525 ~ 0.804), employed status (OR = 2.757, 95%CI = 1.598 ~ 4.756), highest educational level (OR = 0.394, 95%CI = 0.176 ~ 0.880), region (OR = 0.561, 95%CI = 0.437 ~ 0.720) and impact on life by the COVID-19 (OR = 0.482, 95%CI = 0.270 ~ 0.861) were mainly factors associated with residents’ attitudes.

**Conclusions:**

The aims of this study were to evaluate the residents attitudes to information disclosure timely during the low transmission period in China and to provide a scientific basis for effective information communication in future public health crises. Timely and effective efforts to disclose information need to been made during the low transmission period. Continued improvements to local authority reporting will contribute to more effective public communication and efficient public health research responses. The development of protocols and the standardization of epidemic message templates—as well as the use of uniform operating procedures to provide regular information updates—should be prioritized to ensure a coordinated national response.

**Supplementary Information:**

The online version contains supplementary material available at 10.1186/s12889-022-12804-x.

## Background

In December 2019, an outbreak of COVID-19, caused by infection with SARS-CoV-2, emerged in Wuhan, Hubei Province, China, and subsequently became a global pandemic, seriously threatening the lives and health of the public and jeopardizing the stable economic development and social safety of nations [1, 2]. In the absence of effective treatment or a vaccine, the success or failure of mitigating COVID-19 transmission in the population relies heavily on the effectiveness of social distancing, self-protection, case detection, quarantine, isolation, and testing [3]. The effectiveness of these nonpharmaceutical interventions depends on the active participation and engagement of residents in the community, which is substantially influenced by the information disclosure of COVID-19. Given the short disease transmission doubling time [4], the timing (and hence time lags) in the communication of critical public health information to the community has a profound impact on the outcome of public adherence to nonpharmaceutical measures and, ultimately, on the outcome of outbreak mitigation [5]. Only when people feel they have received timely disclosure will they have sufficient incentive to implement community prevention and control measures [6, 7].

Public health emergencies, especially outbreaks of new infectious diseases, are often accompanied by uncertainty about the cause of the emergency. However, the resulting information on the morbidity and mortality of the diseases becomes the focus of public concern from the moment it emerges. Simultaneously, the unknown causes of public health emergencies stimulate increased information-seeking behavior in people who are aiming to reduce their uncertainties about the emergent situation [8]. However, in the absence of information, people experience a wide range of emotions in the face of unexpected situations, and the anxiety or fear thus generated can exacerbate the occurrence [9]. In addition to the professional measures of epidemic prevention and control, keeping the information accurate and transparent are critical parts of the crisis response, reflecting the significance of the establishment of government monitoring-feedback-intervention mechanisms in public health emergencies [8]. Prompt information disclosure is a top priority for preparedness and it enables a collective response to the COVID-19 pandemic [10]. The emergence of The COVID-19 infodemic further reveals the importance of providing timely but also accurate, simple, and non-contradictory information [11, 12]. As the World Health Organization (WHO) declared the COVID-19 outbreak a public health emergency of international concern, people have the right to be clearly informed about the health risks that they and their communities face [13]. The public’s feedback to the timeliness of information disclosure is an important way to reflect the government’s information dissemination work. To help the general public and researchers bridge knowledge gaps and respond to the crisis in a timely manner, whether information has been disclosed timely by the China government was a crucial question.

Since the severe acute respiratory syndrome (SARS) emergency in 2003 and the avian influenza A (H7N9) epidemic in 2013, the Chinese government’s provision of rapid, effective, and efficient disclosure of epidemic information has substantially improved [13, 14]. Starting on January 3, 2020, information regarding COVID-19 cases has been reported to the WHO and the general public through China’s National Health Commission on a daily basis [15]. The time interval from the first case description to the identification of the pathogen on January 7—as well as the availability of probes for PCR detection on January 21—was much faster for COVID-19 than for SARS [16]. The National Health Commission took prompt public health measures and soon classified COVID-19 as a new notifiable disease under the National Infectious Disease Law and the Frontier Health and Quarantine Law on January 20, 2020, thus authorizing by law the prompt disclosure of information about the epidemic at the subnational level [15]. During the epidemic, people expressed high satisfaction with the timeliness of information disclosure [17, 18].

Disclosing information timely and having community-based approaches have been shown to play a significant role in addressing the issues brought about by the COVID-19 outbreak in China. Previous global studies have confirmed the residents’ concern about the epidemic information [19], and some studies have examined the impact of information disclosure on authenticity screening [20]. Some studies have focused on channels [21] and countermeasures [22], but there is no research to observe the timeliness of information disclosure from the perspective of residents. Currently COVID-19 epidemic has been controlled and China has entered a period of low transmission [23]. At this stage, China government still need to disclose information timely. Whether the residents consent they have timely access to COVID-19 information is worth to survey in China. As more and more countries enter the low-transmission phase, maintaining prevention awareness among the disclosing information timely is critical to preventing a secondary outbreak. By investigating the whether people consent that information is disclosed timely and influencing factors of different people’s attitudes towards disclosure in China, we can reflect the prevention awareness of the government and residents during the low transmission period of COVID-19, which can provide a reference for Chinese and global public health policy makers.

## Methods

### Ethics statement

This study protocol was approved by the institutional review board of Tongji Medical College of Huazhong University of Science and Technology, Wuhan, China. All methods were performed in accordance with the relevant guidelines and regulations. Respondents were informed that their participation was voluntary, and consent was implied on the completion of the questionnaire.

### Study participants and survey design

A cross-sectional survey was conducted in China from 4 to 26 February 2021. We stratify the respondents mainly according to the eastern, central and western region of China. We selected residents from eastern (Beijing, Tianjin, Hebei, Liaoning, Shanghai, Jiangsu, Zhejiang, Fujian, Shandong, Guangdong and Hainan), central (Shanxi, Jilin, Heilongjiang, Anhui, Jiangxi, Henan, Hubei, and Hunan) and western (Chongqing, Sichuan, Guizhou, Yunnan, Tibet, Shaanxi, Gansu, Qinghai, Ningxia, Xinjiang, Inner Mongolia, and Guangxi) China to complete the survey. Convenient sampling strategy was adopted to recruit participators; the research team used WeChat (the most popular social media platform in China) to advertise and circulate the survey link to their network members. Network members were requested to distribute the survey invitation to all their contacts. The participants were informed that their participation was voluntary, and consent was implied through their completion of the questionnaire. The inclusion criteria were that the respondents were Chinese citizens who were at least 18 years old, and able to comprehend and read Chinese.

### Instruments

The questionnaire consisted of two parts: 1) socio-demographic characteristics, with 8 items, including gender, age, marital status, highest educational level, place of residence, religion, employment status and have a chronic disease (diagnosed by a doctor); and 2) the recognition to COVID-19 information disclosing timely, with 3 items, including “consent COVID-19 information has been disclosed timely”, “the primary way to get information about the COVID-19” and “COVID-19 have a large impact on your life”. (Supplement questionnaire format).

Wenjuanxing (www.wjx.cn), a widely used platform for conducting surveys in China, was used to develop the electronic questionnaire. An online poster with an access code or the website link to the questionnaire was distributed via two ways: (1) posted on our WeChat; and (2) distributed via WeChat groups, with an average of one to two RMB each as compensation. Each individual could only participate once on each WeChat account to avoid repeated submissions.

### Statistical methods

The data were analysed using *SPSS™* for Windows, Version 22.0 (SPSS, Inc., Chicago, IL, USA). We dichotomous the answers to the “Consent COVID-19 information has been disclosed timely” of residents’ attitudes as “Yes” and “No”. The descriptive statistics were presented as the number of observations with percentage (%), and we analyzed the difference in demographic statistics by Chi-square (χ2) test. Shapiro-Wilk test is used to judge the normality of quantitative data. Due to the disparities in socioeconomic status in different regions, the data have a typical hierarchical structure. The verification of specific assumptions of the logistic regressions (e.g., low multicollinearity, independence of errors, linearity in the logit for continuous variables, and lack of strongly influential outliers) were tested [24]. We performed a mixed-effect logistic regression model with a random cluster effect (geographic regions) to investigate the adjusted OR (95% CI) of influencing factors of residents’ attitudes to agree they acquire adequate community protection. Further, we explored the factors influencing participants’ attitudes in Eastern, Central, and Western China, respectively, through multivariable logistic regression analysis. *P*-values are more suitable as a measure of the strength of evidence against the null hypothesis [25]. Therefore, we talked about degrees of evidence instead of significance and non-significance (e.g., *P*=. 049 and *P*=. 051 are similar results). In the classification of evidence in our study, we divided it into two types: “high” and “low”.

## Results

### Descriptive statistics

A total of 2453 residents received the questionnaire, of which 21 participants did not respond and 71 questionnaires were not filled. The response rate was 96.24% and 2361 complete questionnaires were employed for results analysis. Table 1 reports the social-demographic characteristics of 2361 respondents. The median age was 34 years (Interquartile range = 26–42) and majority of respondents were female (60.10%). Among the respondents, 421 (17.83%), 1470 (62.26%), and 470 (19.91%) were from eastern, central, and western China, respectively. Most respondents (89.24%) have the bachelor’s degree or higher. More than half of the participants (57.05%) were unemployed.

**Table 1:** Statistical description of study samples: univariate Analysis of the differences of residents’ attitudes to consent COVID-19 information has been disclosed timely during the low transmission period

Variables	N (%)	***χ2***	***P***	Evidence Degrees
Total	2361 (100)	NA	NA	NA
Consent COVID-19 information has been disclosed timely				
Yes	1704 (72.17)	NA	NA	NA
No	657 (27.83)			
Gender				
Male	942 (39.90)	0.415	0.520	Low
Female	1419 (61.10)			
Age group, y				
18–44	1845 (78.14)	64.094	**< 0.001**	High
45–59	369 (15.63)			
> 60	111 (4.70)			
Highest educational level				
Primary school or below	68 (2.88)	13.475	0.001	High
Middle school	186 (7.88)			
College degree or above	2107 (89.24)			
Place of residence				
Urban	1372 (58.11)	5.762	**0.016**	High
Rural	989 (41.89)			
Region				
Eastern China	421 (17.83)	35.886	**< 0.001**	High
Central China	1470 (62.26)			
Western China	470 (19.91)			
Employment status				
Employed	1014 (42.95)	8.721	**0.003**	High
Unemployed	1347 (57.05)			
Have a chronic disease (diagnosed by a doctor)				
Yes	1621 (68.66)	0.093	0.761	Low
No	740 (31.34)			
Marital status				
Unmarried	1560 (66.07)	5.929	**0.015**	High
Married	801 (33.93)			
The primary way to get information about the COVID-19				
Traditional Media (Television, Radio and Newspapers)	1361 (57.65)	0.001	0.980	Low
Emerging media (Weibo, WeChat and Interent news)	1000 (42.35)			
COVID-19 have a large impact on your life				
Strongly disagree	620 (26.26)	7.825	0.098	Low
Disagree	964 (40.83)			
Not sure	562 (23.80)			
Agree	124 (5.25)			
Strongly agree	91 (3.85)			

Of them, 1704 (72.17%) consented COVID-19 information has been disclosed timely. The Variance Inflation Factor (VIF) value among the variables was less than five, indicating that there is low multicollinearity. Durbin-Waston test shows that the DW value is 1.67 (close to 2), which verifies the independence of errors. Since all variables are categorical in this study, linearity in the logit for continuous variables is not considered. In addition, significant outliers are eliminated by k-means algorithm. Results of the analysis suggested the gender, age, place of residence, region, employment status and marital status were high statistical significance influencing factors for “consenting COVID-19 information has been disclosed timely” (Table 1). Considering the significant differences different geographic regions in the sampling, we conducted further analyses with participants from Eastern, Central, and Western China, respectively (Table 2). In the mixed-effect logistic regression analysis, Chinese residents who were older (OR = 0.093, 95%CI = 0.043 ~ 0.201), lived in urban (OR = 0.650, 95%CI = 0.525 ~ 0.804), lived in central China (OR = 0.561, 95%CI = 0.437 ~ 0.720), and strongly agreed that COVID-19 have a large impact on your life (OR = 0.482, 95%CI = 0.270 ~ 0.861) were not more likely to consent COVID-19 information has been disclosed timely (Table 3).

**Table 2:** Univariate analysis of the differences in attitudes to consent COVID-19 information has been disclosed timely among the included residents stratified by geographic characteristics

Variables	Eastern China				Central China				Western China			
	N (%)	***χ2***	***P***	Evidence Degrees	N (%)	***χ2***	***P***	Evidence Degrees	N (%)	***χ2***	***P***	Evidence Degrees
Total	421 (100)	NA	NA	NA	1470 (100)	NA	NA	NA	470 (100)	NA	NA	NA
Consent COVID-19 information has been released timely.												
Yes	277 (65.80)	NA	NA	NA	1124 (76.46)	NA	NA	NA	303 (64.47)	NA	NA	NA
No	144 (34.20)				346 (23.54)				167 (35.53)			
Gender												
Male	181 (42.99)	5.126	0.024	High	558 (37.96)	3.937	0.047	High	203 (43.19)	0.048	0.826	Low
Female	240 (57.01)				912 (62.04)				267 (56.81)			
Age group, y												
18–44	323 (76.72)	18.673	**< 0.001**	High	1175 (79.93)	18.164	**< 0.001**	High	347 (73.83)	44.103	**< 0.001**	High
45–59	61 (14.49)				232 (15.78)				76 (16.17)			
> 60	37 (8.79)				63 (4.29)				47 (10.00)			
Highest educational level												
Primary school or below	17 (4.04)	1.302	0.522	Low	27 (1.84)	8.786	**0.012**	High	24 (5.11)	2.555	0.279	Low
Middle school	24 (5.70)				123 (8.37)				39 (8.30)			
College degree or above	380 (90.26)				1320 (89.80)				407 (86.60)			
Place of residence												
Urban	288 (64.41)	8.912	0.003	High	754 (51.29)	15.953	**< 0.001**	High	330 (70.21)	3.396	0.065	Low
Rural	133 (31.59)				716 (48.71)				140 (29.79)			
Employment status												
Employed	270 (64.13)	0.006	0.940	Low	508 (34.56)	3.078	0.079	Low	236 (50.21)	38.391	**< 0.001**	High
Unemployed	151 (35.87)				962 (65.44)				234 (49.79)			
Have a chronic disease (diagnosed by a doctor)												
Yes	286 (67.93)	3.773	0.052	Low	985 (67.01)	0.012	0.912	Low	350 (74.47)	0.647	0.421	Low
No	135 (32.07)				485 (32.99)				120 (25.53)			
Marital status												
Unmarried	222 (52.73)	1.491	0.222	Low	1061 (72.18)	4.981	**0.026**	High	277 (58.94)	24.806	**< 0.001**	High
Married	199 (47.27)				409 (27.82)				193 (41.06)			
The primary way to get information about the COVID-19												
Traditional Media (Television, Radio and Newspapers)	238 (56.53)	0.249	0.618	High	850 (57.82)	0.377	0.539	High	273 (58.09)	0.153	0.696	High
Emerging media (Weibo, WeChat and Interent news)	183 (43.47)				620 (42.18)				197 (41.91)			
COVID-19 have a large impact on your life												
Strongly disagree	103 (24.47)	7.361	0.118	Low	395 (26.87)	2.569	0.632	Low	122 (25.96)	9.503	**0.050**	High
Disagree	181 (42.99)				596 (40.54)				187 (39.79)			
Not sure	105 (24.94)				342 (23.27)				155 (24.47)			
Agree	16 (3.80)				82 (5.58)				26 (5.53)			
Strongly agree	16 (3.80)				55 (3.74)				20 (4.26)

**Table 3:** Mixed-Effect Logistic Regression Analysis on the Influencing Factors of Residents’ attitudes to consent COVID-19 information has been disclosed timely during the low transmission period of the COVID-19

Variables	Coefficient	***S.E.***	***P***	Evidence Degrees	***OR***	95% ***CI***
Gender (Ref: Female)						
Male	0.109	0.097	0.262	Low	1.115	0.922 ~ 1.350
Age group, y (Ref: 18–44)						
45–59	− 0.648	0.144	**< 0.001**	High	0.523	0.394 ~ 0.694
> 60	−2.378	0.394	**< 0.001**		0.093	0.043 ~ 0.201
Highest educational level (Ref: Primary school or below)						
Middle school	−0.202	0.306	0.509	Low	0.817	0.448 ~ 1.489
College degree or above	−0.403	0.266	0.129	Low	0.668	0.397 ~ 1.125
Place of residence (Ref: Rural)						
Urban	−0.431	0.109	**< 0.001**	High	0.650	0.525 ~ 0.804
Region (Ref: Eastern China)						
Central China	−0.578	0.128	**< 0.001**	High	0.561	0.437 ~ 0.720
Western China	0.132	0.147	0.370	Low	1.141	0.855 ~ 1.523
Employment status (Ref: Unemployed)						
Employed	0.232	0.134	0.083	Low	1.261	0.970 ~ 1.639
Have a chronic disease (diagnosed by a doctor) (Ref: No)						
Yes	−0.084	0.103	0.414	Low	0.92	0.752 ~ 1.124
Marital status (Ref: Unmarried)						
Married	0.12	0.135	0.375	Low	1.128	0.865 ~ 1.471
The primary way to get information about the COVID-19 [Ref: Traditional Media (Television, Radio and Newspapers)]						
Emerging media (Weibo, WeChat and Interent news)	0.008	0.096	0.932	Low	1.008	0.835 ~ 1.217
COVID-19 have a large impact on your life (Ref: Strongly disagree)						
Disagree	−0.164	0.117	0.162	Low	0.849	0.674 ~ 1.068
Not sure	−0.125	0.133	0.347	Low	0.883	0.681 ~ 1.145
Agree	−0.209	0.239	0.381	Low	0.811	0.508 ~ 1.296
Strongly agree	−0.729	0.296	**0.014**	High	0.482	0.270 ~ 0.861

In addition, we stratified the study sample by regions and conducted multivariate logistic regression analyses. Male (OR = 1.396, 95%CI = 1.085 ~ 1.797) and employed (OR = 2.757, 95%CI = 1.598 ~ 4.756) were also the factors associated with an increased willingness to consent COVID-19 information has been disclosed timely in Central China and Western China respectively; while the highest educational level is college degree or above (OR = 0.394, 95%CI = 0.176 ~ 0.880) was a deduced factor in Central China (Table 4).

**Table 4:** Stepwise Multivariate Logistic Regression Analysis on the Influencing Factors of Residents’ attitudes to consent COVID-19 information has been disclosed timely during the low transmission period of the COVID-19

Variables	Coefficient	***S.E.***	***P***	Evidence Degrees	***OR***	95% ***CI***
**Eastern China**						
Age group, y (Ref: 18–44)						
> 60	−2.336	0.75	0.002	High	0.097	0.022 ~ 0.420
Place of residence (Ref: Rural)						
Urban	−0.698	0.259	0.007	High	0.498	0.299 ~ 0.828
**Central China**						
Gender (Ref: Female)						
Male	0.334	0.129	0.009	High	1.396	1.085 ~ 1.797
Age group, y (Ref: 18–44)						
45–59	−0.416	0.185	0.025	High	0.660	0.459 ~ 0.948
> 60	−1.826	0.600	0.002	High	0.161	0.050 ~ 0.522
Highest educational level (Ref: Primary school or below)						
College degree or above	−0.932	0.41	0.023	High	0.394	0.176 ~ 0.880
Place of residence (Ref: Rural)						
Urban	−0.431	0.14	0.002	High	0.65	0.494 ~ 0.856
**Western China**						
Age group, y (Ref: 18–44)						
45–59	−1.155	0.348	0.001	High	0.315	0.159 ~ 0.623
> 60	−2.722	0.749	< 0.001	High	0.066	0.015 ~ 0.286
Employment status (Ref: Unemployed)						
Employed	1.014	0.278	< 0.001	High	2.757	1.598 ~ 4.756
COVID-19 have a large impact on your life (Ref: Strongly disagree)						
Strongly agree	−2.157	1.068	0.043	High	0.116	0.014 ~ 0.939

## Discussion

Our study is based on a cross-sectional survey, determined the residents’ attitudes to consent COVID-19 information has been disclosed timely and influencing factors. We found that 1704 (72.17) of residents consent COVID-19 information has been disclosed timely during the low transmission period of the COVID-19. Furthermore, age, gender, place of residence, employed status, highest educational level, region and impact on life by the COVID-19 were mainly factors associated with residents’ attitudes.

COVID-19 disproportionately impacts vulnerable populations [26]. Access to health information has long been limited for many people, and access barriers and health disparities are likely exacerbated during this pandemic [27]. Older, female, unemployed and those impacted by COVID-19 expressed concern about the lack of timely information disclosing in this study. In fact, these groups were vulnerable in the COVID-19 pandemic and therefore need more adequate information support, which suggested that the government should pay more attention to ensuring the information of vulnerable groups in the process of information disclosure. On the other hand, residents who live in urban and have college degree or above showed higher expectations for information disclosure. A possibility is that these people are more aware of the importance of disclosure, and therefore more eager to get full information. This finding also provides further evidence for the relationship between cognitive level and health needs.

Varieties of information dissemination channels make it easier for people to obtain the information. In addition to the traditional way of disseminating information by word of mouth, we can also get information from online social networks, news websites and television stations, to name only a few [28]. Another finding of our study is that access to information does not affect residents’ perceptions of the timeliness of information disclosure. This may be due to the emergence of multiple types of media, people are getting used to getting information from multiple sources and placing the same importance on these information. The epidemic spreading can trigger epidemic-relevant information to disseminate among the population [29] and the government should use a variety of channels to disclose information, promote the popularization of information.

When a new infectious disease breaks out, residents, especially where the epidemic is more severe, may bear heavier psychological stress [30, 31]. Some studies showed that psychological health problem among residents in Wuhan, where the epidemic was most severe, were higher than elsewhere,and were strongly correlated with the severity of the epidemic and closely mirrored the number of new cases per day [32, 33]. Our study reported that residents in central China expressed more concern about the timeliness of information disclosure. One possible reason is that COVID-19 outbreaks have mainly occurred in central China, especially in Hubei province, where residents have a greater demand for information disclosure. This further illustrates the profound impact of the pandemic on society, namely, the increased demand for government information disclosure and, based on this, the awakening of higher expectations for public health and government services.

### Strengths and limitations

This study is the first study reported residents’ attitudes to consent COVID-19 information has been disclosed timely and influencing factors,which can reflect awareness of prevention COVID-19 during the low transmission period. We used a nationwide sample of the Chinese population. This study provided a scientific basis for effective information communication in future public health crises, which are of importance to China and other countries.

However, this study has some limitations. First, this study used social media as the main method to disseminate the survey. Participants without access to the internet were probably not included. Second, the distribution of the study participants was imbalanced across regions (421: 1470: 470); therefore, the subgroups of variables might not be representative of the population. Third, this study could not determine how many participants reviewed the online poster or survey but decided not to complete the survey; thus, the presence of non-response bias could not be assessed. Finally, as the behaviors were self-reported, reporting bias was possible. This study is based on a cross-sectional survey and cannot prove a clear causal relationship. In addition, it should also be noted that the intention to stay informed does not necessarily correspond to the predisposition to take action. Indeed, phenomena such as pandemic fatigue reduce compliance with anti-pandemic norms [34]. Overall, generalization of the results should be regarded with caution.

## Conclusions

Promoting the timely disclosure of information related to public health emergencies and providing the public with regular channels through which authoritative up-to-date information is disclosed are both essential for the timely communication of risk information and guidance. This study reported that 1704 (72.17) of residents consent COVID-19 information has been disclosed timely during the low transmission period of the COVID-19. Furthermore, residents who were female, older, unemployed, lived in urban and central China, impacted by COVID-19, and had college degree or above expressed stronger concerns about the timeliness of information disclosure.

Our results augment the awareness of information and data disclosed timely during the low transmission period of the COVID-19 in China. Therefore, the development of uniform protocols and standards of epidemic message templates must be encouraged, as should the use of standard operating procedures to regularly update all vital information in a manner that the public can easily interpret and researchers can effectively analyze. As more and more countries enter the low-transmission phase, this study suggest that these issues should be considered a critical policy priority for the national health authorities in China and most countries worldwide.

## Supplementary Information

**Additional file 1.**

## Data Availability

Data may be made available by contacting the corresponding author.
