# Supplementary material for: Timeliness of information disclosure during the low transmission period of COVID-19: resident-level observational study in China
Source: BMC Public Health. 2022 Mar 1;22:415. doi: 10.1186/s12889-022-12804-x (PMC8887935; doi:10.1186/s12889-022-12804-x)
Supplement: Supplementary file 1 — Additional file 1. [file 12889_2022_12804_MOESM1_ESM.docx]

**Supplement questionnaire format**

1.Gender

①Male

②Female

2.Age______

3.Highest educational level

①Primary school or below

②Middle school

③College degree or above

4.Place of residence

①Urban

②Rural

5.Region

①Eastern China

②Central China

③Western China

6.Employment status

①Employed

②Unemployed

7.Have a chronic disease (diagnosed by a doctor)

①Yes

②No

8.Marital status

①Unmarried

②Married

9.The primary way to get information about the COVID-19

①Traditional Media (Television, Radio and Newspapers)

②Emerging media (Weibo, WeChat and Interent news)

10.COVID-19 have a large impact on your life

①Strongly disagree

②Disagree

③Not sure

④Agree

⑤Strongly agree

11.Do you Consent COVID-19 information has been disclosed timely

①Yes

②No
